# Side-by-side comparison of the effects of Gq- and Gi-DREADD-mediated astrocyte modulation on intracellular calcium dynamics and synaptic plasticity in the hippocampal CA1

**DOI:** 10.1186/s13041-021-00856-w

**Published:** 2021-09-20

**Authors:** Yana Van Den Herrewegen, Thomas M. Sanderson, Surajit Sahu, Dimitri De Bundel, Zuner A. Bortolotto, Ilse Smolders

**Affiliations:** 1 Department of Pharmaceutical Chemistry, Drug Analysis and Drug Information, Research Group Experimental Pharmacology, Center for Neurosciences (C4N), Vrije Universiteit Brussel (VUB), Laarbeeklaan 103, 1090 Brussels, Belgium; 2 School of Physiology, Pharmacology and Neuroscience, University of Bristol, Tankard’s Cl, University Walk, BS8 1TD Bristol, UK

**Keywords:** Astrocytes, Chemogenetics, DREADDs, Calcium imaging, Long-term potentiation, LTP, Hippocampus, Schaffer collateral

## Abstract

Astrocytes express a plethora of G protein-coupled receptors (GPCRs) that are crucial for shaping synaptic activity. Upon GPCR activation, astrocytes can respond with transient variations in intracellular Ca^2+^. In addition, Ca^2+^-dependent and/or Ca^2+^-independent release of gliotransmitters can occur, allowing them to engage in bidirectional neuron-astrocyte communication. The development of designer receptors exclusively activated by designer drugs (DREADDs) has facilitated many new discoveries on the roles of astrocytes in both physiological and pathological conditions. They are an excellent tool, as they can target endogenous GPCR-mediated intracellular signal transduction pathways specifically in astrocytes. With increasing interest and accumulating research on this topic, several discrepancies on astrocytic Ca^2+^ signalling and astrocyte-mediated effects on synaptic plasticity have emerged, preventing a clear-cut consensus about the downstream effects of DREADDs in astrocytes. In the present study, we performed a side-by-side evaluation of the effects of bath application of the DREADD agonist, clozapine-N-oxide (10 µM), on Gq- and Gi-DREADD activation in mouse CA1 hippocampal astrocytes. In doing so, we aimed to avoid confounding factors, such as differences in experimental procedures, and to directly compare the actions of both DREADDs on astrocytic intracellular Ca^2+^ dynamics and synaptic plasticity in acute hippocampal slices. We used an adeno-associated viral vector approach to transduce dorsal hippocampi of male, 8-week-old C57BL6/J mice, to drive expression of either the Gq-DREADD or Gi-DREADD in CA1 astrocytes. A viral vector lacking the DREADD construct was used to generate controls. Here, we show that agonism of Gq-DREADDs, but not Gi-DREADDs, induced consistent increases in spontaneous astrocytic Ca^2+^ events. Moreover, we demonstrate that both Gq-DREADD as well as Gi-DREADD-mediated activation of CA1 astrocytes induces long-lasting synaptic potentiation in the hippocampal CA1 Schaffer collateral pathway in the absence of a high frequency stimulus. Moreover, we report for the first time that astrocytic Gi-DREADD activation is sufficient to elicit de novo potentiation. Our data demonstrate that activation of either Gq or Gi pathways drives synaptic potentiation through Ca^2+^-dependent and Ca^2+^-independent mechanisms, respectively.

## Introduction

Astrocytes are one of the most abundant resident cells of the brain, well known for providing metabolic support, ensuring homeostasis, and maintaining the blood–brain barrier. Astrocytes express a wide array of G protein-coupled receptors (GPCRs), allowing them to respond to synaptic activity. Indeed, astrocytes have emerged as key players at quad-partite synapses and were found to actively participate in bidirectional neuron-astrocyte communication via transient variations in intracellular Ca^2+^ [1, 2] and the release of gliotransmitters, such as glutamate, γ-aminobutyric acid (GABA), and/or adenosine triphosphate (ATP)/adenosine (reviewed by [3, 4]). Astrocytic Ca^2+^ signaling generally remains a complex and poorly understood concept [2] and is often dysregulated in various CNS disorders [5–9]. Therefore, it is of utmost importance to understand how astrocytes tune neuronal activity and modulate higher brain functions, as this can aid in the discovery of potential novel therapeutic strategies for multiple brain disorders. It is well-established that increases in astrocytic Ca^2+^ transients can be elicited by activation of Gq-coupled GPCRs [3, 10–13]. This astrocytic Gq-GPCR activation recruits a similar downstream intracellular cascade as observed in response to Gq-GPCR activation in neurons. Briefly, astrocytic Gq-GPCR activation recruits the Gαq protein, which in turn leads to 1,4,5-trisphosphate (IP_3_)-mediated Ca^2+^ release from internal stores [13]. In contrast, although there is evidence of a role of Gi-GPCR signalling in astrocytes, the nature of the signalling has been less consistently reported in the literature. Canonically, Gi-GPCR activation recruits the G_αi/o_ protein which inhibits adenylate cyclase and thus, reduces intracellular cyclic adenosine monophosphate (cAMP), while the Gβγ subunit activates inwardly rectifying K^+^ channels and/or inhibits Ca^2+^ channels [14]. Indeed, a reduction in cAMP has been reported upon Gi-GPCR activation in astrocytes [15], suggesting that these receptors are coupled to the expected G_αi/o_ protein. Regarding the effect on intracellular Ca^2+^, either no effect on Ca^2+^ levels [16] or decreased baseline Ca^2+^ levels, presumed to be the resulting action of the Gβγ subunit [17], have been reported after Gi-GPCR activation in hippocampal [16] and ventral midbrain astrocytes [16, 17], respectively. However there is also ample evidence showing an increase in astrocytic Ca^2+^ transients upon Gi-GPCR activation, such as activation of GABA_B_Rs and dopamine D1 and D2-type receptors in the hippocampus [18–21], GABA_B_Rs in the somatosensory cortex [22], GABA_B_Rs in the ventral tegmental area [23] and the µ-opioid receptor (MOR) and dopamine D1 receptors in the nucleus accumbens [24, 25]. Moreover, these GABA_B_R and MOR-induced increases in cytosolic Ca^2+^ were suggested to be derived from intracellular stores [19, 22, 24, 26], as these Ca^2+^ transients were shown to be abolished in inositol 1,4,5-trisphosphate receptor type 2 (IP_3_R2) knock-out mice [19, 22, 24] or when IP_3_-gated internal Ca^2+^ stores were depleted [19, 26]. The underlying mechanisms mediating astrocytic Ca^2+^ transients via Gi-GPCR activation remain elusive. In addition, activation of either Gq- or Gi-GPCRs on astrocyte surfaces impact synaptic transmission and plasticity via either Ca^2+^-dependent [10, 19, 27–31] or Ca^2+^-independent gliotransmitter release [16, 32]. Interestingly, the magnitude and temporal characteristics of astrocyte-mediated synaptic modulation, were found to be significantly affected by the pattern of interneuron activity, that caused GABA release and in turn stimulated hippocampal astrocytic GABA_B_Rs [33]. This means that astrocytes can interpret and integrate time- and frequency-related variations in stimulation and, thus suggests that differences in experimental protocols related to GPCR agonist application, such as duration and concentration, could potentially complicate correct interpretation of the obtained results.

Unfortunately, further investigation of reciprocal neuron-astrocyte interactions have long been hindered due to the lack of selective tools to modulate astrocytic GPCR signalling [34, 35]. Designer receptors exclusively activated by designer drugs (DREADDs) are an interesting tool to tackle these issues as they use GPCR-mediated intracellular signal transduction pathways naturally present in astrocytes. DREADDs have been increasingly used to study effects on intracellular GPCR-dependent astrocytic Ca^2+^ signalling and on synaptic transmission and/or plasticity. Similar to endogenous GPCRs, Gq- and Gi-coupled DREADDs have both been shown to induce Ca^2+^ responses in astrocytes [36–42]. However, while Gq-DREADD-induced astrocyte modulation is consistently reported to increase astrocytic Ca^2+^ events or baseline Ca^2+^ levels [36–42], the effect of Gi-DREADDs on astrocytic Ca^2+^ signalling remains less clear [39, 40, 43]. Differences in intracellular Ca^2+^ responses upon Gi-DREADD mediated astrocyte modulation have been reported, not only between brain regions [40], but also within the same region i.e., hippocampus [39, 40, 43]. More specifically, application of the DREADD agonist, clozapine-N-oxide (CNO), to hippocampal slices transduced with Gi-DREADD has been shown alternately to elicit transient increases in intracellular Ca^2+^ [39], to decrease intracellular baseline Ca^2+^ levels [43] or to have no discerable effect [40].

To date, only a limited amount of evidence has been gathered regarding gliotransmitter release upon Gq- or Gi-DREADD activation in hippocampal astrocytes. For instance, slow inward currents (SICs) in surrounding neurons were observed upon CNO bath application in hippocampal slices expressing either astrocytic Gq-DREADD or Gi-DREADD [39], which is typically attributed to the activation of extrasynaptic neuronal *N*-methyl-d-aspartate receptors (NMDARs) via astrocytic released glutamate [44]. Interestingly, Chai et al. [40] did not observe any changes in SIC amplitude or frequency, nor did they observe increased glutamate release using a genetically encoded glutamate sensor (iGluSnFR) upon CNO bath application in Gq-DREADD transduced hippocampal astrocytes. In addition, the release of d-serine has been implied upon astrocytic Gq-DREADD activation [36]. It is however accepted that activation of both Gq- and Gi-coupled DREADDs can alter hippocampal synaptic plasticity [16, 36, 39, 43]. For instance, Gq-DREADD-mediated astrocytic activation was found to induce long-term potentiation (LTP) in the *Cornu Ammonis 1* (CA1) of the hippocampus without any additional, sub-threshold electrical stimulus [36], while Gi-DREADD activation was proposed to reduce LTP threshold, as LTP was induced solely upon application of an additional 40-Hz stimulus [16].

In this research paper we compare in parallel Gq- and Gi-DREADD-mediated activation of hippocampal astrocytes and evaluate their effects on intracellular Ca^2+^ dynamics in astrocytes and synaptic plasticity in the hippocampal CA1 Schaffer collateral pathway. In addition, we emphasize the importance of reporting the application duration and concentration of DREADD agonists, to allow proper comparison of the effects on astrocytic Ca^2+^ and synaptic plasticity in ex vivo slices.

## Materials and methods

### Animals

All experiments were performed on male C57BL/6J mice (Charles River Laboratories, UK), 8 weeks old at the time of surgery. All mice were group-housed in a temperature (19–23 °C) and humidity (30–70% relative humidity) regulated environment with a 12/12 h light/dark cycle and received food pellets and water ad libitum. Mice were habituated 1 week to the animal house upon arrival and were subsequently subjected to surgery. All procedures involving animals were conducted in accordance with the Animal Scientific Procedures Act 1986, UK, and with approval of the University of Bristol. To the best of our abilities, results were reported in accordance with the ARRIVE guidelines [45].

### Viral vector transduction

Astrocyte-specific expression of the DREADDs was obtained using an adeno-associated viral (AAV) vector expressing either the Gq- or Gi-DREADD fused to the red fluorescent protein mCherry under the GFAP promotor (Gq-DREADD: vector AAV8-GFAP-hM3Dq-mCherry, diluted 1:50 with sterile saline with a final concentration of 2.96 × 10^12^ gc/ml, plasmid #50478 acquired from Addgene; Gi-DREADD: vector AAV8-GFAP-hM4Di-mCherry, diluted 1:50 with sterile saline with a final concentration of 2.46 × 10^12^ gc/ml, plasmid #50479 acquired from Addgene); or the control vector AAV8-GFAP-mCherry (undiluted 2.7 × 10^12^ vg/ml, obtained from UNC Vector Core by Dr. Boyden).

Mice were anesthetized with isoflurane and received s.c. 2 mg/kg lidocaine (2% Xylocaine^®^ Solution injectable, Aspen, UK), s.c. 5 mg/kg carprofen (50 mg/ml, Rimadyl^®^ Small Animal Solution for Injection, Pfizer, USA) and 0.9% NaCl (Baxter) to assure appropriate pain killing and hydration. After disinfection of the skin with Isobetadine^®^, an incision was made from between the eyes until the end of the skull. Using a stereotactic microinjection system, bilateral hippocampal infusion of an AAV vector (500 nl per injection site) was performed at the following coordinates relative to Bregma (anteroposterior (AP), − 1.5 mm, mediolateral (ML), ± 1 mm, dorsoventral (DV), − 1.6 mm; site 2: AP − 2.5 mm, ML ± 2 mm, DV − 1.6 mm). The syringe was maintained in place for an additional 10 min. to limit back flow along the injection track. After injection, the skin was closed using nonabsorbable suturing material (Ethilon II, 4-0, M-2, Ethicon). Mice were group housed and sacrificed for ex vivo experiments 3–5 weeks after surgery to ensure full expression of the DREADDs.

### Chemicals

CNO, a DREADD agonist, was acquired from Enzo LifeSciences (Belgium). CNO is dissolved in DMSO at a concentration of 30 mM. Stock solution is diluted to reach a final working concentration of 10 µM in artificial cerebrospinal fluid (aCSF). Oregon Green™ 488 BAPTA-1 acetoxymethyl ester (OGB-1 AM) was obtained from ThermoFischer Scientific (USA). The Ca^2+^-dye was prepared as described in [46]: to each 50 µg-vial of the dye, 3.87 µl of dimethyl sulfoxide (DMSO) and 9 µl of a 20% (w/v) Pluronic F-127 (ThermoFischer Scientific, Rockford, USA) was added and was maintained in an ultrasound bath for 15 min. Right before dye-loading, this solution was mixed with 100 µl artificial cerebrospinal fluid (aCSF) and filtered.

### Hippocampal slice preparation

Acute coronal hippocampal slices were prepared from 11–13 weeks old male, C57Bl6/J mice, previously injected with viral vectors driving DREADD-hM3Dq or DREADD-hM4Di or control vector expression in their astrocytes. All mice were killed by cervical dislocation according to Schedule 1 of the United Kingdom (Scientific Procedures) Act of 1986. Brains were rapidly removed and placed into ice-cold sucrose-containing aCSF (87 mM NaCl, 75 mM sucrose, 25 mM NaHCO_3_, 2.5 mM KCl, 1.25 mM NaH_2_PO_4_, 7 mM MgCl_2_, 0.6 mM ascorbic acid, 0.5 mM CaCl_2_, and 25 mM d-glucose) oxygenated with 95% O_2_ and 5% CO_2_. Slices were cut using a vibratome (Leica VT1200).

### Ex vivo* calcium *imaging

**Fig. 1:**
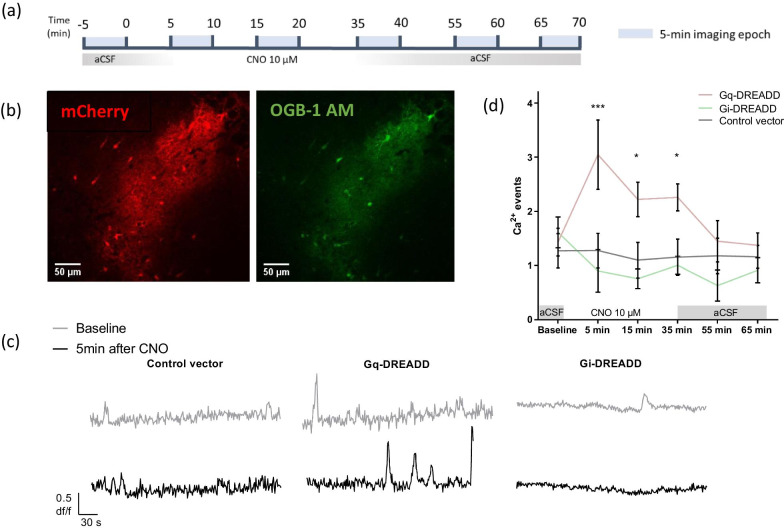
Spontaneous Ca^2+^ events during 5-min periods following long-term chemogenetic manipulation of astrocytes. **a** Timeline overview of the experimental procedure. **b** Two-photon confocal image of CA1 astrocytes expressing DREADDs, fused to an mCherry protein for visualization (red) and the fluorescent Ca^2+^ indicator OGB-1 AM (green). Scale bar of 50 µm is marked in white. **c** Representative traces of individual astrocytes before and 5 min after CNO application. Graph (**d**) shows the average amount of Ca^2+^ events per 5-min imaging periods recorded from CA1 astrocytes before, during and after CNO (10 µM) application. RM Two-Way ANOVA shows a significant difference between groups. Bonferroni’s post-hoc analysis shows significant differences in the Gq-DREADD transduced mice 5 to 35 min after CNO application compared to control transduced slices (control vector vs. Gq-DREADD; 5 min after CNO p = 0.0006; 15 min after CNO, p = 0.0377; 35 min after CNO, p = 0.0410; Gq-DREADD, n = 5; control vector, n = 4), while no significant differences were observed for the Gi-DREADD during nor after CNO application (Bonferroni’s post-hoc analysis, control vector vs. Gi-DREADD; 5 min after CNO p = 0.8973; 15 min after CNO, p = 0.9766; 35 min after CNO, p > 0.9999; 20 min after wash-out, p = 0.5380; 30 min after wash-out, p > 0.9999; n = 4). *p < 0.05; ***p < 0.001. Data are represented as the mean ± SEM, Gq-DREADD, n = 5; Gi-DREADD, n = 4; and control vector, n = 4

Coronal 300-µm sections were incubated for 45 min at 35 °C in a holding chamber with continuously oxygenated low Ca^2+^-aCSF (124 mM NaCl, 26 mM NaHCO_3_, 3 mM KCl, 1.4 mM NaH_2_PO_4_, 6 mM MgSO_4_, 1 mM CaCl_2_, and 12 mM d-glucose; ± 310 mOsm) and afterwards kept for two additional hours at room temperature in normal aCSF (124 mM NaCl, 26 mM NaHCO_3_, 3 mM KCl, 1.4 mM NaH_2_PO_4_, 1 mM MgSO_4_, 2 mM CaCl_2_, and 10 mM d-glucose). Slices were then transferred to a submerged recording chamber (28 °C), continuously perfused with aCSF (2 ml/min), and bolus loading of Oregon Green BAPTA-1 AM calcium dye was performed. A glass pipette filled with the dye solution was lowered into the *stratum radiatum* of the slice, and the dye was ejected twice (5 psi) for 3 min. at two locations 100 µm from each other. After dye loading, slices were allowed to rest for an additional 30 min, enabling intracellular dye-uptake. Images were acquired at 1.1 frame/s using a Zeiss LSM510 two-photon confocal microscope. The imaging protocol was based on [36]; shortly, after 5-min baseline recording, CNO (10 μM) was bath-applied for a total duration of 35 min. During this period, 5-min recordings were alternated with 5-min ‘rest’ periods to avoid photo bleaching. After 35 min, CNO was washed-out and imaging was paused for 20 min. Subsequently alternated imaging re-started for an additional period of 30 min (Fig. 1a).

Ca^2+^-imaging analysis was performed using ImageJ (NIH) and changes in fluorescence intensity were recorded by drawing analysis boxes over individual astrocytic cell bodies. Ca^2+^-events were defined if peak amplitude was larger than 3 standard deviations (SD) above the mean of average baseline fluorescence for 2 consecutive data points or more (∆F/F0).

### Ex vivo slice electrophysiology

**Fig. 2:**
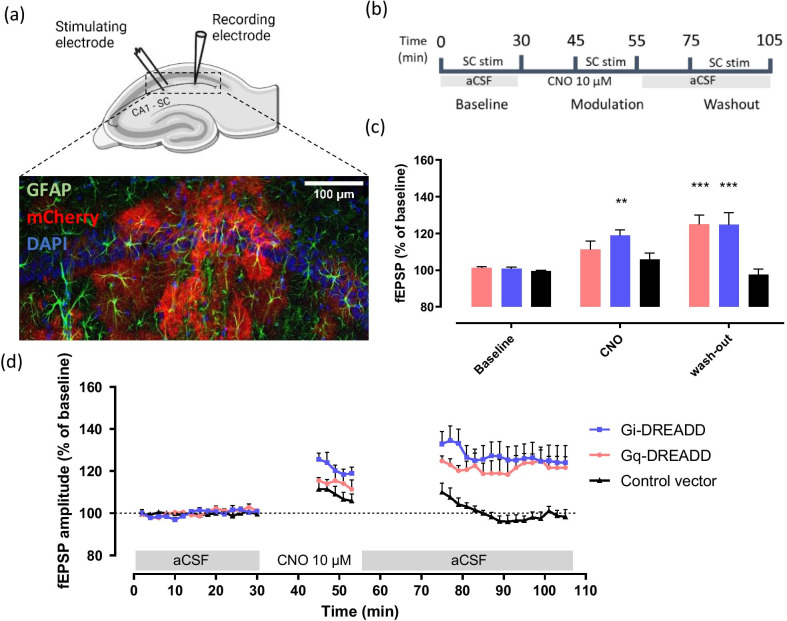
The effect of Gq- and Gi-DREADD activation in hippocampal astrocytes on plasticity of CA1 synapses. **a** Schematic overview of the electrode positions during CA1-Schaffer collateral stimulation (positioned 0.5 mm apart, created with BioRender.com). Immunohistochemistry showed CA1 astrocytes (GFAP, green) expressing DREADDs, which are fused to an mCherry protein (mCherry, red) and cell nuclei (DAPI, blue). Scale bar of 100 µm is marked in white. **b** Schematic timeline of the experimental procedure. Graph (**c**) shows the average fEPSP amplitude of the last stimulation in every phase of the experiment (baseline, CNO and wash-out). Comparisons between baseline and CNO or wash-out were made for every group using RM Two-Way ANOVA Bonferroni’s Post-hoc analysis, and significant results are illustrated on the graph with an asterisk, **p < 0.01; ***p < 0.001. **d** The graph shows the amplitude of the fEPSPs recorded from Schaffer Collateral (SC) stimulation in CA1 in time upon CNO (10 µM) application. Data are represented as the mean ± SEM, Gq-DREADD, n = 4; Gi-DREADD, n = 4; and control vector, n = 5

Extracellular field recordings were performed in CA1 following [36]. Briefly, 400-µm coronal slices were incubated for 30 min. at 35 °C and then kept for 1 h at room temperature in a holding chamber with oxygenated normal aCSF (124 mM NaCl, 26 mM NaHCO_3_, 3 mM KCl, 1.4 mM NaH_2_PO_4_, 1 mM MgSO_4_, 2 mM CaCl_2_, and 10 mM d-glucose). Slices were then transferred to a submerged recording chamber (28 °C). Extracellular synaptic activity was measured using a bipolar tungsten electrode to deliver stimuli (frequency of 0.033 Hz) to the Schaffer collateral pathway, evoking field excitatory postsynaptic potentials (fEPSPs) recorded from a 3 M NaCl-containing glass microelectrode (3–5 MΩ) in the stratum radiatum of CA1 (Fig. 2a). Amplitude of fEPSPs were recorded on-line using the WinLTP software [47]. After obtaining stable responses, slices were incubated in aCSF containing 10 μM CNO for 25 min, allowing evaluation of the effect of CNO on synaptic plasticity (Fig. 2b). The amplitude of fEPSPs in CA1 was monitored for at least 30 min following wash-out. All slices were viewed under the Zeiss LSM510 confocal fluorescent microscope to verify DREADD-mCherry expression.

### Statistics

Analyses, statistical evaluation and presentation of the data were performed with Prism v7.0 (GraphPad Software, Inc.). Multiple groups of data were analysed by Repeated Measures (RM) Two-Way analysis of variance (ANOVA) followed by the Bonferroni test for post-hoc comparisons. All tests were performed with α = 0.05. p values < 0.05 were considered significant. All results are expressed as means ± SEM.

## Results

### Gq-DREADD, but not Gi-DREADD, activation in hippocampal astrocytes induces transient increases in astrocytic Ca^2+^ events

To investigate long-term effects (35 min) on astrocytic Ca^2+^-transients exerted by activation of either Gq- or Gi-coupled DREADDs by the designer drug CNO, ex vivo two-photon Ca^2+^ imaging was performed. To this end, hippocampal slices from C57BL/6J mice were used, that had been previously injected with viral vectors driving expression of either Gq-DREADDs, Gi-DREADDs or mCherry (in case of control viral vector) in GFAP^+^ cells. The effect on spontaneous astrocytic Ca^2+^ events during 5-min imaging epochs before, during and after CNO (10 µM, 35 min) bath application was assessed (Fig. 1a), using OGB-1 AM as fluorescent Ca^2+^ indicator (Fig. 1b).

RM Two-Way ANOVA showed a significant interaction (F (10, 50) = 3.995, p = 0.0005, n = 4–5), a significant time effect (F (5, 50) = 3.224, p = 0.0134, n = 4–5), and a main DREADD effect (F (2, 10) = 4.294, p = 0.0451, n = 4–5). Specifically, after 5-min CNO bath application, Gq-DREADD-expressing astrocytes showed elevated intracellular Ca^2+^ transients (3.05 ± 0.64, n = 5) (Fig. 1c). Significant increases in Ca^2+^ events were observed throughout the entire duration of CNO application in Gq-coupled-DREADD-expressing slices (Fig. 1d, Bonferroni’s post-hoc analysis, control vector vs. Gq-DREADD; 5 min after CNO p = 0.0006; 15 min after CNO, p = 0.0377; 35 min after CNO, p = 0.0410; n = 4–5) and only returned to baseline after wash-out (Fig. 1d, Bonferroni’s post-hoc analysis, control vector vs. Gq-DREADD; 20 min after wash-out, p > 0.999; 30 min after CNO, p > 0.999; n = 4–5). CNO did not significantly affect spontaneous Ca^2+^ events in Gi-DREADD-expressing astrocytes (0.90 ± 0.39, n = 4) nor control vector transduced astrocytes (1.27 ± 0.32, n = 4) (Fig. 1c). Importantly, in Gi-DREADD-expressing slices no significant difference in astrocytic Ca^2+^ events were recorded during CNO application nor after wash-out, compared to slices transduced with the control vector (Fig. 1d, Bonferroni’s post-hoc analysis, control vector vs. Gi-DREADD; 5 min after CNO p = 0.8973; 15 min after CNO, p = 0.9766; 35 min after CNO, p > 0.9999; 20 min after wash-out, p = 0.5380; 30 min after wash-out, p > 0.9999; n = 4). Our data shows that bath application of 10 µM CNO for 35 min elicits robust, spontaneous increases in Ca^2+^ transients in Gq-DREADD-expressing astrocytes, which return to baseline after changing the perfusion medium to CNO-free, normal aCSF, while Gi-DREADDs trigger a Ca^2+^-independent intracellular pathway upon activation.

### Both Gq- and Gi-DREADD activation in hippocampal astrocytes induces potentiation at hippocampal CA1 synapses in absence of high frequency stimulation

Subsequently, we examined the effect of either astrocytic Gq- or Gi-DREADD-mediated modulation on synaptic transmission at Schaffer collateral neuronal synapses in area CA1 (Fig. 2a). More specifically, using extracellular field potential recordings, we monitored the effect on low frequency (0.033 Hz) evoked synaptic events before, during and after CNO (10 µM) bath application, as previously described for Gq-DREADD activation [36]. In short, after obtaining stable baseline responses, the DREADD agonist CNO (10 µM) was applied to the bath and recordings were stopped for 15 min. Next, 0.033 Hz Schaffer collateral stimulation and field recordings resumed for 10 min during continuous CNO perfusion. Finally, perfusion medium was switched to CNO-free, normal aCSF to allow wash-out of CNO and recordings were paused again, only to be resumed 20 min after (Fig. 2b).

RM Two-Way ANOVA showed a significant interaction (F (4, 30) = 5.427, p = 0.0021; n = 4–5), a significant time effect (F (2, 30) = 15.00, p < 0.0001; n = 4–5) and a DREADD effect (F (2, 30) = 14.24, p < 0.0001; n = 4–5). Strikingly, in our set-up, during CNO application, we observed a significant increase in fEPSP amplitude for Gi-DREADD expressing slices compared to baseline (Fig. 2c, d; Post-hoc Bonferroni’s multiple comparisons test, Gi-DREADD baseline vs. CNO p = 0.0047). As expected, fEPSP amplitude for control vector transduced slices during CNO application did not differ significantly compared to baseline (Fig. 2c, d; Post-hoc Bonferroni’s multiple comparisons test; control baseline vs. CNO p = 0.3738). Yet, a small transient increase in amplitude of fEPSPs in control transduced slices can be observed after resuming stimulation, which is commonly reported upon interruption of evoked potentials [48]. In addition, fEPSP elicited in Gi-DREADD expressing hippocampal slices differed significantly from control transduced slices during CNO application (Fig. 2c, d; Post-hoc Bonferroni’s multiple comparisons test; Gi-DREADD CNO vs. control CNO p = 0.0371). For Gq-DREADD expressing slices, a trend towards increased fEPSP amplitude during CNO application was observed compared to baseline (Fig. 2c, d; Post-hoc Bonferroni’s multiple comparisons test, Gq-DREADD baseline vs. CNO p = 0.1383). Interestingly, both Gq-DREADD (125.08 ± 4.96%, n = 4) and Gi-DREADD (124.69 ± 6.77%, n = 4) expressing hippocampal slices, but not control slices (97.45 ± 3.13%, n = 5), exhibited significant potentiation after changing the perfusion medium to CNO-free, normal aCSF, which was long-lasting (> 30 min) (Fig. 2c, d Post-hoc Bonferroni’s multiple comparisons test, Gq-DREADD baseline vs. wash-out p = 0.0002; Gi-DREADD baseline vs. wash-out p = 0.0002; control baseline vs. wash-out p = 0.8961).

As such, our data suggests that activation of either Gq- or Gi-coupled DREADDs on astrocytes is able to induce long-term (> 30 min) synaptic potentiation at hippocampal CA1 neuronal synapses in the absence of high frequency stimulation and that activation of the Gi-coupled DREADDs is sufficient to induce de novo potentiation.

## Discussion

In the past few years, chemogenetic approaches have been used to evaluate the crucial role of astrocytes in higher brain functions and this has drastically broadened our understanding of these star-like cells. However, proficient comprehension of the intracellular processes and subsequent effects on the surrounding neuropil are imperative to fully grasp their contribution in physiological and pathological conditions. Ongoing research on this topic unveiled astrocyte-mediated effects on synaptic plasticity [16, 36], but also, several inconsistencies on astrocytic Ca^2+^ signalling [39, 40, 43] preventing unambiguous characterization of the downstream effects of the DREADDs.

In the present study, we performed a side-by-side comparison of astrocyte activation using the two most commonly used DREADDs, hM3Dq (Gq protein-coupled GPCR) and hM4Di (Gi protein-coupled GPCR), and studied their effects on intracellular astrocytic Ca^2+^-signalling and synaptic plasticity in the hippocampal CA1 neuronal network. We showed that 35-min application of DREADD agonist CNO (10 µM), induced a robust increase in intracellular astrocytic Ca^2+^ events in Gq-DREADD, but not Gi-DREADD, expressing CA1 hippocampal astrocytes compared to control vector transduced astrocytes. In addition, we showed that both Gq- as well as Gi-mediated astrocyte modulation successfully potentiated synaptic transmission in the Schaffer collateral pathway, which was long-lasting and in the complete absence of any additional high-frequency stimulation.

Our results show that long-term Gq-DREADD activation in astrocytes resulted in a robust long-lasting increase in Ca^2+^ events, which is in line with the literature [36] and expectations with regard to the well-known downstream signalling upon Gq-coupled GPCRs. In addition, after switching bath perfusion from CNO-containing aCSF to CNO-free, normal aCSF, the amount of Ca^2+^ transients returned to baseline, suggesting wash-out of the DREADD agonist. However, we cannot exclude that other mechanisms, such as desensitization of the Gq-DREADD, occur, causing this decrease in Ca^2+^ transients. Interestingly, upon astrocytic Gi-DREADD activation, different effects on astrocytic Ca^2+^ have been previously described. For instance, Kol et al. [43] found that Gi-DREADD activation (10 min, 10 µM) induced a slight decrease in baseline Ca^2+^ levels of hippocampal astrocytes, while Durkee et al. [39] reported increases in intracellular Ca^2+^ upon puff application of CNO (2–5 s, 1 mM) in Gi-DREADDs expressing hippocampal astrocytes. In addition, Chai et al. [40] did not find consistent increases in intracellular Ca^2+^ hippocampal astrocytes during CNO bath application (4 min, 1 µM). Likewise, we observed no changes in the frequency of Ca^2+^ transients of Gi-DREADD transduced slices following DREADD agonism (35 min, 10 µM). Although, the mechanisms involving such variations on intracellular Ca^2+^ are hitherto still unknown, we propose that these discrepancies might be ascribed to a difference in astrocyte stimulation intensity, i.e. combination of the concentration and duration of the Gi-GPCR agonist application. Intriguingly, recently individual hippocampal astrocytes were found to be able to release both ATP/adenosine and glutamate and this in a time-dependent and activity-sensitive manner upon GABA released during interneuron activity [33]. This suggests that the type and intensity of astrocyte stimulation is crucial in determining which downstream signalling pathway is induced by astrocytic GABA_B_R activation. However, whether this differentiation in downstream signalling pathway upon GABA_B_R activation affects Ca^2+^ responses and if this is the case for Gi DREADDs as well, remains to be investigated. Nonetheless, astrocytic Gi-DREADD modulation has been shown to inhibit astrocytic cAMP signalling [49, 50], thus it is reasonable to assume that the hM4Di-DREADD receptor is coupled to the G_αi/o_ protein, but that the βγ subunits of the G protein act differently upon activation depending on the intensity of stimulation. Interestingly, Gi-DREADD-induced Ca^2+^ elevations in hippocampal astrocytes upon short-term, high concentration CNO application, were recently shown to be mediated via direct binding of the βγ subunits to IP_3_R2 [39].

Next, we investigated the effects of either Gq-DREADD or Gi-DREADD-mediated astrocyte activation on synaptic plasticity in the Schaffer collateral-CA1 hippocampal network. As experimental protocols and slicing conditions can affect the characteristics of cells and networks and, thus experimental outcomes, we lay out the details of the methods provided in two papers focusing on DREADD-based LTP induction [16, 36] in comparison to our experimental procedures, in Table 1. Upon Gq-DREADD activation in hippocampal astrocytes, we observed LTP in the absence of any high frequency electrical stimulus, as previously reported [36]. Significant potentiation of fEPSP responses occurred following the wash-out phase of the DREADD agonist CNO. This is in contrast to observations made by Adamsky et al. [36], who reported a greater magnitude of potentiation that was apparent immediately during the first stimulation after CNO application. This discrepancy may be explained by differences in experimental set-up (see Table 1). For example, we prepared our slices in sucrose containing cutting solution, a manipulation that has previously been shown to reduce the magnitude of LTP [51].

**Table 1:** Methodological specifications of ex vivo field recordings in the Schaffer collateral pathway, using Gq- and Gi-DREADD based astrocyte modulation

	Current study, Van Den Herrewegen et al. (2021)	Adamsky et al. [36]	Nam et al. [16]
*Viral vectors and used chemogenetic tools*			
Viral vector with DREADD construct	*Gq-DREADD: AAV8-GFAP-hM3Dq-mCherry *Gi-DREADD: AAV8-GFAP-hM4Di-mCherry *Control vector: AAV8-GFAP-mCherry	*Gq-DREADD: AAV8-GFAP-hM3Dq-mCherry *Gi-DREADD: / *Control vector: AAV8-GFAP-mCherry	*Gq-DREADD: / *Gi-DREADD: AAV-GFAP-hM4Di-mCherry *Control vector: AAV-GFAP-GFP
*Animals*			
Mouse strain/age	C57Bl6J/11–13 weeks	C57Bl6J/11–13 weeks	C57Bl6J/7–8 weeks
Gender	Male	Male	Male
Group/single housed	Group	Group	Ns
*Slicing conditions*			
Slice orientation and thickness	Coronal 400 µm	Coronal 400 µm	Transverse 350–400 µm
Slicing solution (in mM)	75 Sucrose, 87 NaCl, 2.5 KCl, 25 NaHCO_3_, 1.25 NaH_2_PO_4_, 7 MgCl_2_, 0.5 CaCl_2_, 0.6 Ascorbic acid and 25 glucose	126 NaCl, 2.6 KCl, 26 NaHCO_3_, 1.25 NaH_2_PO_4_, 1 MgCl_2_, 0.625 CaCl_2_ and 10 glucose	212.7 sucrose, 5 KCl, 26 NaHCO_3_, 1.23 NaH_2_PO_4_, 10 MgSO_4_, 0.5 CaCl_2_, and 10 dextrose
Slice recovery conditions	1 h submerged	1 h submerged	1 h ns
*DREADD agonist*			
DREADD agonist	CNO	CNO	CNO
Concentration	10 µM	10 µM	ns
Duration of CNO application	25 min	25 min	10 min
*Slice electrophysiology recording conditions*			
Stimulation frequency	0.033 Hz	0.017 Hz	0.07 Hz
Stimulations during baseline	30 min (60 stimulations)	10 min (10 stimulations)	10 min (40 stimulations)
Pause in stimulation	Yes	Yes	No
Stimulations necessary for induction of synaptic potentiation	Gq-DREADD: 21 Gi-DREADD: 1	Gq-DREADD: 1 (1 mM Mg^2+^) 21 (2 mM Mg^2+^)	Gi-DREADD: 40+ additional 40 Hz stimulus
Bath temperature	28 °C	32 °C	28–30 °C
aCSF composition (in mM)	124 NaCl, 3 KCl, 26 NaHCO_3_, 1.4 NaH_2_PO_4_, 1 MgSO_4_, 2 CaCl_2_ and 10 glucose	126 NaCl, 2.6 KCl, 26 NaHCO_3_, 1.25 NaH_2_PO_4_, 1 MgCl_2_, 2 CaCl_2_ and 10 glucose	124 NaCl, 5 KCl, 26 NaHCO_3_, 1.23 NaH_2_PO_4_, 1 MgSO_4_, 2 CaCl_2_ and 10 dextrose

*ns* not specified

The most novel finding of our study is the fact that we show for the first time that Gi-DREADD activation of astrocytes induces de novo long-lasting potentiation, as significant increases in fEPSP amplitude were already observed from the first stimulation after CNO application onwards, which lasted longer than 30 min after wash-out. Previously, Gi-DREADD activation of hippocampal astrocytes was shown to reduce the threshold of LTP induction, as LTP was elicited when 40-Hz stimulation was performed in addition to CNO bath application [16]. However, in our study we showed that low-frequency stimulation (0.033 Hz) is sufficient to evoke LTP. This might be ascribed to variations in astrocytic GPCR stimulation, i.e. duration and concentration of GPCR agonist application, as previously discussed, which can affect outcomes on synaptic plasticity [33]. In addition, other experimental differences, for instance, slice recovery conditions, such as submerging of the slice, in combination with Mg^2+^ concentrations are critical players in permitting metaplastic effects on LTP duration [52], which were not fully disclosed in [16]. Moreover, Ca^2+^ uncaging in astrocytes, has also been reported to reduce LTP threshold, and only to induce LTP in combination with an additional depolarization [31]. However, as no effect on astrocytic Ca^2+^ transients were observed upon Gi-DREADD activation, our data suggests that Gi-DREADD activation of astrocytes affects synaptic plasticity in Ca^2+^-independent manner.

Astrocytes actively shape synaptic transmission via release of neuroactive substances, such as gliotransmitters (glutamate, GABA, ATP, adenosine, and d-serine), synaptogenic cues (e.g. thrombospondins, tumor necrosis factor alpha) and metabolic substrates (e.g. lactate, lipids) (reviewed by [53]). The context-specific release of gliotransmitters is regulated via activation of GPCRs, which in turn can induce enhanced Ca^2+^ transients (reviewed by [54]). Indeed, multiple endogenous astrocytic Gq-GPCRs and Gi-GPCRs have been reported to elicit increases in Ca^2+^ transients, followed by a release of active substances and/or gliotransmitters [10, 18, 19, 22, 24, 27–29, 31, 55]. Likewise, activation of the exogenous Gq-DREADDs expressed in hippocampal astrocytes, was previously proposed to induce synaptic potentiation via Ca^2+^-dependent release of d-serine [36]. The underlying mechanisms of GPCR-induced Ca^2+^-dependent gliotransmitter release can be divided in two major categories: vesicular exocytosis, relying on soluble *N*-ethylmaleimide-sensitive factor attachment protein receptor (SNARE) complexes [56, 57] and non-vesicular release via gliotransmitter-permeable ion channels (such as opening of the Ca^2+^-regulated Bestrophin-1 channel [58, 59]). Besides Ca^2+^-dependent release of gliotransmitters, there is some evidence showing that activation of astrocytic GPCRs is able to trigger Ca^2+^-independent release of gliotransmitters. Particularly, several Gi-GPCRs, such as the µ-opioid receptor (MOR) [16, 60], GABA_B_ receptor, cannabinoid receptor 1, and adenosine 1 receptor [58], have been shown to trigger glutamate release via a Ca^2+^-independent mechanism upon activation. This Ca^2+^-independent release of glutamate was found to rely on the interaction of the dissociated Gβγ complex with the glutamate-permeable, TWIK-related K^+^ (TREK-1) channel i.e., a two-pore domain potassium channel [58]. Intriguingly, such Ca^2+^-independent mediated glutamate release has been shown to affect synaptic plasticity. More specifically, astrocytic MOR activation, co-localized with and known to release glutamate via the TREK-1 channel [60], was shown to induce LTP in the presence of an additional high frequency (40-Hz) stimulus [16]. This effect was ascribed to activation of presynaptic metabotropic glutamate receptors 1 (mGluRs1) and, notably, could be successfully mimicked by activation of Gi-DREADDs in astrocytes [16]. However, also other mediators can be released independent of increases in Ca^2+^ transients. For instance, activation of astrocytic mGluR3, a Gi-GPCRs, was recently shown to release prostaglandin E_2_ (PGE_2_) in the presence of a blocker of the endoplasmic reticulum Ca^2+^ pump (cyclopiazonic acid). Moreover, they showed that this astrocytic PGE_2_ release led to altered synaptic plasticity, i.e. enhanced mEPSPs in surrounding neurons [61]. Noteworthy, peripheral astrocytic processes (PAPs) are critically involved in synaptic plasticity and are known to display spotty Ca^2+^ transients in their microdomains [27, 62, 63]. Although no elevations in Ca^2+^ transients were observed in our study, assessment of Ca^2+^ transients was restricted to the soma and large processes, as Ca^2+^-imaging was performed using two-photon Ca^2+^ imaging after bolus loading of a chemical dye (OGB-1 AM), which is known to have limited spatial resolution [64–66]. Imaging of PAPs typically requires high-resolution and time-consuming imaging techniques, such as 3D two-photon Ca^2+^ imaging in combination with genetically-encoded Ca^2+^ indicator [64] or electron microscopy [66]. Therefore, we cannot fully exclude that there is no Ca^2+^-dependent process in the PAPs involved in the Gi-DREADD-mediated synaptic plasticity in our experiments. Nevertheless, taken together, these findings emphasize the possibility of Gi-GPCR-mediated Ca^2+^-independent release of active substances, which in turn can affect synaptic plasticity. Particularly, Gi-GPCR-mediated Ca^2+^-independent glutamate release via the two-pore domain potassium TREK-1 channel is the most promising mechanism for the observed Ca^2+^-independent Gi-DREADD-induced de novo potentiation. Notwithstanding, additional data should be gathered to verify if this Gi-DREADD-induced synaptic potentiation is Ca^2+^-independent.

Our present results suggest that Gq- and Gi-DREADD, based astrocyte modulation, rely on different mechanisms to induce LTP. This postulation is reinforced by the different responses observed during long-term Ca^2+^ signalling in Gq- and Gi-DREADDed astrocytes. Activation of Gq-DREADDs expressed in astrocytes was previously proposed to induce NMDAR-dependent LTP formation via the release of d-serine [36]. More specifically, slices pre-treated with 10 μM d-serine, failed to induce synaptic potentiation upon additional CNO-mediated Gq-DREADD activation, likely due to occlusion [36]. Moreover, another study showed that clamping of intra-astrocytic Ca^2+^ levels suppressed LTP at nearby synapses, which was rescued by adding 10 μM d-serine [29]. Taken together, this indicates Ca^2+^-dependent d-serine from astrocytes release is crucial for LTP induction. On the contrary, previous research showed that activation of Gi-DREADDs in astrocytes lowered the threshold for LTP induction, which was ascribed to astrocytic glutamate release and the subsequent activation of neuronal presynaptic metabotropic glutamate receptors 1 (mGluRs 1) [16]. This is in line with the proposed Ca^2+^-independent TREK-1 channel-mediated release of glutamate, as these channels were reported to induce a fast release that facilitates high extracellular peak concentrations of glutamate, necessary to activate mGluRs [58]. Agonism of group I mGluRs (mGluR1 and mGluR5) has been previously reported to facilitate LTP [67–69], however an additional mechanism is required to induce de novo LTP in CA1 synapses [70, 71]. It is therefore likely that the observed astrocytic Gi-DREADD-induced de novo potentiation is evoked via an additional mechanism, besides mGluR agonism. Indeed, astrocyte-mediated glutamatergic signalling has been repeatedly reported to induce LTP via various mechanisms in addition to mGluR activation [3, 10]. Moreover, even though TREK-1 channel-mediated glutamate release predominantly activates mGluRs [16, 58, 60], it was also reported to activate NMDARs [58]. Therefore, it is expected that Gi-DREADD activation in astrocytes induces activation of both glutamate receptors i.e., NMDAR and mGluR, and thus triggers an NMDAR-dependent LTP. Additionally, astrocytes have also been reported to be involved in NMDAR-independent LTP [10]. Specifically, application of acetylcholine induced an NMDAR-independent, cholinergic-LTP in CA3-CA1 synapses. This effect relied on muscarinic acetylcholine receptor (mAChR)-mediated glutamate release from astrocytes, which subsequently activated neuronal mGluRs, in addition to simultaneous depolarization of the postsynaptic neuron [10]. However, in this study, mAChR-mediated glutamate release was shown to rely on increased intracellular Ca^2+^ in the astrocytic processes [10]. In addition, ATP, shown to be co-released with glutamate from individual hippocampal astrocytes [33], is able to further facilitate LTP induction upon group I mGluR and NMDAR co-activation [72]. Notably, a caveat of the current study is that we cannot fully exclude that the small, but not significant, increase in fEPSP amplitude after a break in the stimulation protocol observed in control slices (Fig. 2d), is crucial for NMDAR co-activation together with Gi-DREADD-mediated mGluR1, and thus contributes to Gi-DREADD-mediated potentiation. Nonetheless, it should be further investigated to confirm whether Gi-DREADD activation truly elicits an NMDAR-dependent LTP. Therefore, we encourage future research comparing the effects of both DREADDs on synaptic plasticity and to elucidate the underlying mechanisms. Moreover, we wish to emphasize the importance of reporting detailed descriptions of the experimental procedures to avoid discrepancies.

In summary, we showed that Gq-DREADD activation in astrocytes triggered a robust increase in Ca^2+^ events, which was not observed upon astrocytic Gi-DREADD activation. Importantly, we demonstrated that both Gq-DREADD and Gi-DREADD activation of astrocytes are sufficient to induce long-lasting potentiation of CA1 synapses, even in the absence of high frequency stimulation. In particular, we show that Gi-DREADD activation of hippocampal astrocytes induces de novo potentiation, potentially in a Ca^2+^-independent manner. Further investigation is required to fully characterize the downstream mechanisms involved in the modulation of intracellular Ca^2+^ signalling upon Gi- and Gq-DREADD mediated astrocyte activation. Comprehensive knowledge of astrocytic GPCR downstream signalling will help unveil the various roles of the excitatory and inhibitory neuro- and gliotransmitters in the hippocampal network and the resulting effects on brain function. Moreover, recent research has put astrocytic DREADD modulation forward as a potential therapeutic strategy in the treatment of CNS diseases [49, 73, 74] and understanding the relevant signalling cascades in physiological conditions will be the first step towards translation to the clinic.

## Data Availability

The datasets used and/or analysed during the current study are available from the corresponding author on reasonable request.

## Abbreviations

AAV: Adeno-associated viral vector
aCSF: Artificial cerebrospinal fluid
ATP: Adenosine triphosphate
CA1: *Cornu Ammonis* 1
cAMP: Cyclic adenosine monophosphate
CNO: Clozapine-N-oxide
DREADDs: Designer receptors exclusively activated by designer drugs
fEPSP: Field excitatory postsynaptic potentials
GABA: γ-Aminobutyric acid
GABA_B_R: γ-Aminobutyric acid receptor B
GPCR: G protein-coupled receptor
IP_3_: Inositol 1,4,5-trisphosphate
IP_3_R2: Inositol 1,4,5-trisphosphate receptor type 2
LTP: Long-term potentiation
mGluR: Metabotropic glutamate receptor
NMDA: *N*-Methyl-d-aspartate
NMDAR: *N*-Methyl-d-aspartate receptor
OGB-1 AM: Oregon Green™ 488 BAPTA-1 acetoxymethyl ester
RM Two-Way ANOVA: Repeated measures Two-Way analysis of variance
SD: Standard deviation
SEM: Standard error on the mean
